# Crohn’s Disease Mimicking Behçet’s Disease and Complicated by Colonic Perforation: A Case Report

**DOI:** 10.7759/cureus.104520

**Published:** 2026-03-01

**Authors:** Fahd J Nazir, Syed Sami N Zafar Ahmed, Zahed Barak, Neil K Bansal, Muhammad A Arshad

**Affiliations:** 1 College of Medicine, Touro College of Osteopathic Medicine, Great Falls, USA; 2 Radiology, Avera McKennan Hospital and University Health Center, Sioux Falls, USA; 3 Internal Medicine, Avera McKennan Hospital and University Health Center, Sioux Falls, USA

**Keywords:** anchoring bias, behçet's disease, biologic therapy, colonic perforation, crohn disease, diagnostic dilemma, inflammatory bowel disease, mucocutaneous ulcers, transmural inflammation

## Abstract

Behçet’s disease and Crohn’s disease share overlapping clinical features, including recurrent oral and genital ulcerations, arthralgias, and gastrointestinal inflammation, frequently leading to diagnostic uncertainty. Accurate differentiation is essential, as management strategies and long-term outcomes differ substantially. We report a diagnostically challenging case of a 60-year-old Hispanic male who initially presented with progressive weight loss, painful oral and genital ulcers, arthralgias, and chronic diarrhea and was treated for presumed Behçet’s disease with corticosteroids and immunomodulatory therapy, resulting in partial symptomatic improvement. Despite treatment, his condition deteriorated over several months, culminating in severe abdominal pain and bowel perforation. Computed tomography (CT) revealed pneumoperitoneum and sigmoid wall thickening, prompting emergent exploratory laparotomy with Hartmann’s procedure for feculent peritonitis. Histopathologic examination demonstrated multifocal ulceration with transmural mixed inflammation and serositis without evidence of vasculitis or granuloma formation, representing the key diagnostic turning point and confirming Crohn’s disease over intestinal Behçet’s disease. Postoperatively, surgical pathology confirmed a diagnosis of Crohn’s colitis, for which he was initiated on infliximab with subsequent clinical stabilization. This case highlights the diagnostic challenges posed by overlapping mucocutaneous and gastrointestinal manifestations, underscores the importance of early histologic confirmation and multidisciplinary evaluation, and emphasizes the need for ongoing diagnostic reassessment to prevent catastrophic complications in patients with systemic inflammatory presentations.

## Introduction

Behçet’s disease and Crohn’s disease are chronic inflammatory disorders that may share overlapping clinical manifestations, including recurrent oral and genital ulcerations, arthralgias, and gastrointestinal involvement [1]. This overlap can complicate diagnostic evaluation, particularly when mucocutaneous findings predominate. Although intestinal involvement is recognized in both conditions, distinguishing between Behçet’s disease and Crohn’s disease remains challenging, and misclassification may result in inappropriate therapy and progressive disease. Diagnostic overlap between these diseases has been associated with misclassification and delayed recognition. Such delays may result in prolonged corticosteroid exposure, deferred biologic therapy, and increased risk of severe and rare complications, such as bowel perforation. Behçet’s disease is a systemic vasculitis, whereas Crohn’s disease is characterized by transmural intestinal inflammation and a relapsing course. When inadequately treated, Crohn’s disease carries a substantial risk of severe complications [2]. We report a case of Crohn’s disease initially misdiagnosed as Behçet’s disease in a patient with prominent mucocutaneous symptoms, resulting in delayed diagnosis and colonic perforation. This case highlights the importance of early endoscopic evaluation, histologic confirmation, and multidisciplinary management in atypical inflammatory presentations.

## Case presentation

A 60-year-old Hispanic male with a history of type 2 diabetes mellitus initially presented to his primary care provider with progressive oral ulcerations, odynophagia, arthralgia, papulopustular skin lesions involving the forehead, neck, and chest, and progressive unintentional weight loss. Otolaryngologic evaluation revealed ulceration of the right vallecula and aryepiglottic fold, with biopsy demonstrating inflammatory granulation tissue without dysplasia or malignancy. Based on the presence of recurrent oral ulcerations, genital ulcerations, and papulopustular skin lesions, the patient met the International Criteria for Behçet’s Disease (ICBD). He was referred for outpatient ophthalmologic evaluation to assess for ocular involvement; however, the results of this evaluation were not available. Initial concern was for Behçet’s disease, and he was empirically treated with systemic corticosteroids, colchicine, and apremilast, resulting in partial symptom improvement.

Eight months later, he presented to the hospital with worsening generalized weakness, chronic diarrhea, severe painful oral ulcers, and approximately 80 pounds of unintentional weight loss. On admission, he appeared cachectic and mildly tachycardic, with diffuse abdominal tenderness and multiple aphthous oral ulcers. Laboratory studies revealed anemia, markedly elevated inflammatory markers (erythrocyte sedimentation rate 117 mm/hr, C-reactive protein 25.9 mg/L), and hypoalbuminemia. An extensive infectious evaluation, including stool studies for common bacterial pathogens and *Clostridioides difficile* toxin, as well as testing for cytomegalovirus, human immunodeficiency virus, tuberculosis, and hepatitis, was negative. Autoimmune serologies, including antinuclear antibody and antineutrophil cytoplasmic antibody, were also negative. HLA-B51 testing was negative.

**Figure 1:**
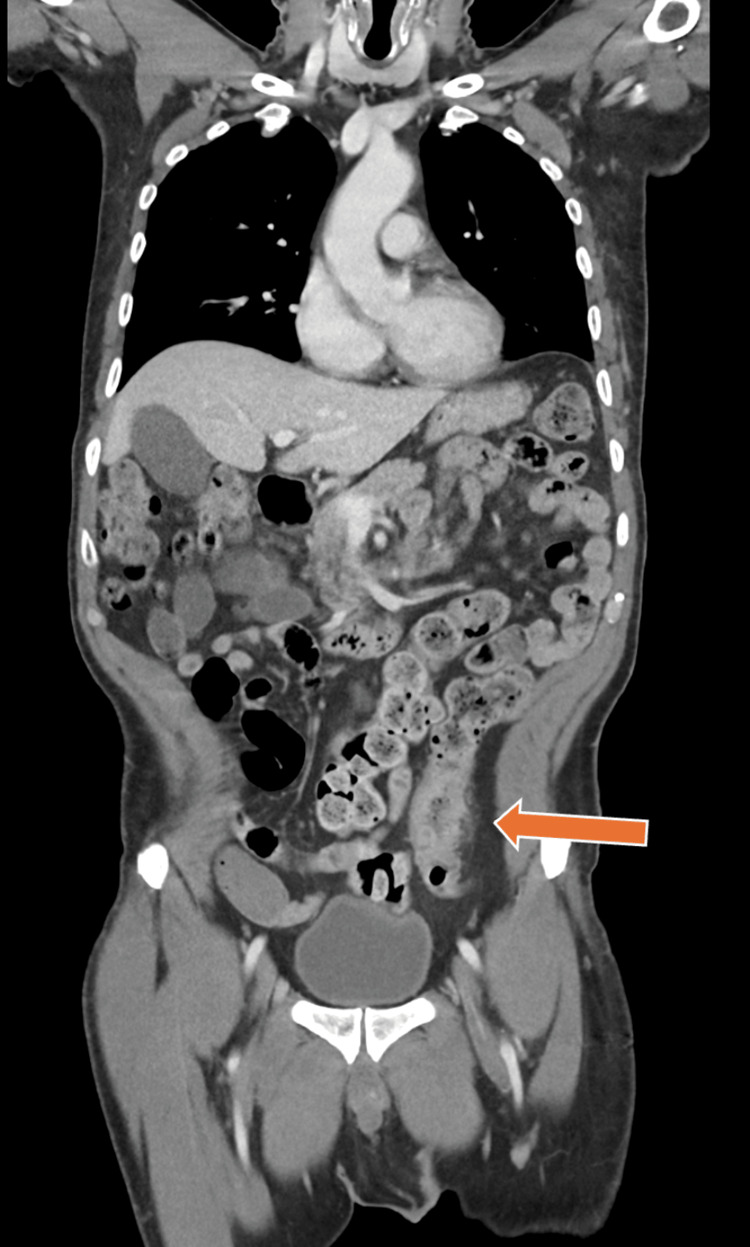
Contrast-enhanced CT of the abdomen and pelvis demonstrating segmental wall thickening and mucosal hyperenhancement of the distal descending and sigmoid colon (arrow), with surrounding inflammatory changes, consistent with active colitis, without evidence of free intraperitoneal air or perforation.

CT of the abdomen and pelvis demonstrated segmental wall thickening with adjacent pericolonic fat stranding involving the distal descending to proximal sigmoid colon with associated marked stool burden. The terminal ileum was spared, and there was no evidence of skip lesions, fistulization, abscess formation, mucosal hyperenhancement, or pathologic adenopathy. Mildly dilated small bowel loops were noted without a transition point. These findings were initially interpreted as stercoral colitis or mild diverticulitis (Figure 1). Colonoscopy revealed patchy severe inflammation with deep, confluent circumferential ulcerations involving the anus, sigmoid, and descending colon, with relative rectal sparing. Biopsies demonstrated active colitis with cryptitis and crypt abscesses, negative for cytomegalovirus, dysplasia, or malignancy. Although the endoscopic findings raised concern for inflammatory bowel disease (IBD), contemporaneous endoscopic assessment noted diagnostic uncertainty and favored Behçet’s disease based on fulfillment of ICBD criteria, prominent mucocutaneous manifestations, and partial response to corticosteroids. Despite continued corticosteroid therapy, his symptoms progressed with worsening abdominal pain and diarrhea.

**Figure 2:**
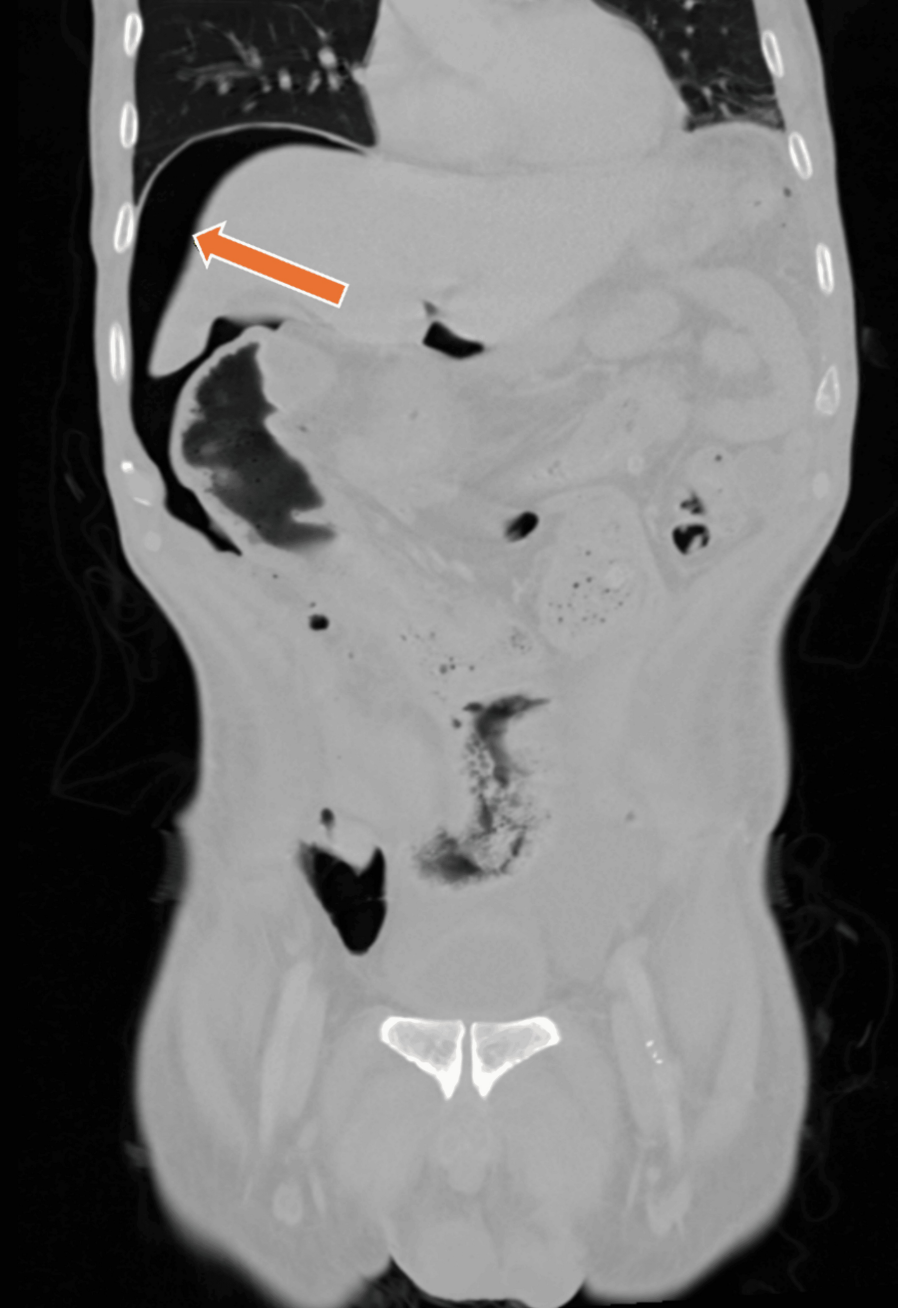
Repeat contrast-enhanced CT of the abdomen and pelvis demonstrating pneumoperitoneum (arrow) with free intraperitoneal air beneath the diaphragm, consistent with bowel perforation and prompting emergent surgical intervention.

During hospitalization, repeat CT imaging demonstrated marked sigmoid wall thickening with pneumoperitoneum, consistent with bowel perforation (Figure 2). He underwent emergent exploratory laparotomy with Hartmann’s procedure, which revealed feculent peritonitis. Postoperatively, he required mechanical ventilation, vasopressor support, and empiric broad-spectrum antimicrobial therapy with intravenous piperacillin-tazobactam, vancomycin, and micafungin for septic shock.

Surgical pathology demonstrated multifocal ulceration with transmural mixed acute and chronic inflammation, necrosis, and serositis without evidence of malignancy, vasculitis, or granuloma formation. Additional sections revealed villous blunting with increased intraepithelial lymphocytes and crypt architectural distortion, consistent with IBD. Given the transmural inflammation, rectal sparing, negative vasculitic workup, progressive disease course, and histopathologic findings, gastroenterology determined that his presentation was most consistent with Crohn’s disease rather than Behçet’s colitis. He was subsequently initiated on infliximab therapy with a corticosteroid taper, resulting in gradual clinical improvement.

## Discussion

Distinguishing Behçet’s disease from Crohn's disease remains clinically challenging due to shared gastrointestinal and systemic features, including oral ulceration, arthralgias, abdominal pain, diarrhea, and gastrointestinal bleeding. Both conditions may involve the terminal ileum, further contributing to diagnostic uncertainty [1-3].

Several studies have examined distinguishing clinical features. In a retrospective analysis comparing 35 patients with intestinal Behçet’s disease and 106 patients with Crohn’s disease, substantial overlap in gastrointestinal symptoms was observed; however, notable differences were identified, with extraintestinal manifestations more prevalent in Behçet’s disease and perianal disease and intestinal obstruction more commonly associated with Crohn’s disease [2]. In our patient, the presence of recurrent oral and genital ulcerations initially favored Behçet’s disease; however, the absence of perianal involvement and the later development of transmural intestinal pathology ultimately aligned more closely with Crohn’s disease.

The two disorders also differ in underlying pathophysiology. Behçet’s disease is a multisystem vasculitis that affects vessels of all sizes, while Crohn’s disease is defined by chronic, transmural granulomatous inflammation [3,4]. Endoscopic findings may further aid differentiation. Behçet’s disease more commonly demonstrates round or oval “punched-out” ulcers with discrete margins and focal distribution patterns in the ileocecal region, whereas longitudinal ulcers, cobblestone appearance, and pseudopolyps are more suggestive of Crohn’s disease. Clinically significant perianal disease and obstructive complications favor Crohn’s disease, while severe gastrointestinal hemorrhage is reported more frequently in Behçet’s disease [2].

Histopathology remains one of the most informative differentiating tools. Crohn’s disease typically shows chronic transmural inflammation, crypt abscesses, and noncaseating epithelioid granulomas [4]. In contrast, Behçet’s disease shows neutrophilic phlebitis without full-thickness bowel wall involvement [1,3]. Although the absence of granulomas does not exclude Crohn’s disease, their presence supports the diagnosis in the appropriate clinical context.

However, despite these characteristic features, misclassification remains common because neither condition is defined by a single definitive diagnostic criterion [5]. In the absence of disease-specific biomarkers and despite the availability of nonspecific inflammatory markers such as fecal calprotectin, recent studies have identified candidate proteomic and metabolomic signatures that may enable more accurate differentiation between these conditions [6].

The ICBD provides a standardized diagnostic framework based on weighted clinical features, including oral aphthosis, genital aphthosis, ocular lesions, skin manifestations, neurologic involvement, and vascular disease. A cumulative score of ≥4 points is considered diagnostic. In this case, the patient initially met ICBD criteria based on recurrent oral ulcerations, genital ulcerations, and papulopustular skin lesions, supporting the initial clinical suspicion for Behçet’s disease. However, although the ICBD criteria facilitate early recognition, they do not reliably distinguish intestinal Behçet’s disease from Crohn’s disease in patients with prominent gastrointestinal involvement, underscoring the need for integration of clinical, endoscopic, and histopathologic data [7].

In the present case, multiple findings supported Crohn’s disease over Behçet’s disease. Surgical pathology demonstrated multifocal ulceration with transmural mixed inflammation and serositis, findings incompatible with a primary vasculitic process. Colonoscopy revealed rectal sparing, a pattern more typical of Crohn’s disease. The disease course was steroid-refractory and progressed to bowel perforation requiring emergent surgery. Importantly, histopathologic examination revealed no evidence of vasculitic features such as neutrophilic phlebitis. Collectively, these features supported Crohn’s disease as the definitive diagnosis and prompted initiation of anti-tumor necrosis factor (anti-TNF) therapy with infliximab.

Cognitive bias plays an important role in complex diagnostic decision-making, particularly in systemic inflammatory diseases with overlapping features. Anchoring bias, in which early diagnostic impressions disproportionately influence subsequent clinical reasoning, may occur when initial findings appear to fit a recognizable disease pattern, as in this case. In conditions such as Behçet’s disease and Crohn’s disease, shared mucocutaneous and gastrointestinal manifestations can obscure the underlying pathology and complicate early diagnostic assessment [8]. In this case, the initial clinical response to corticosteroid therapy may have falsely reinforced a diagnosis of Behçet’s disease. However, corticosteroids exert broad anti-inflammatory effects and may transiently improve symptoms across a wide range of inflammatory conditions, including IBD [9]. As a result, short-term symptomatic improvement is nonspecific and does not reliably distinguish between these entities, and should not preclude continued diagnostic evaluation when clinical features evolve.

Progressive disease despite corticosteroid therapy, marked weight loss, and worsening gastrointestinal symptoms prompted further investigation, ultimately revealing transmural intestinal inflammation and bowel perforation. These findings underscore the importance of ongoing diagnostic reassessment when patients fail to respond as expected or develop new or worsening features inconsistent with the initial working diagnosis. Early reconsideration of alternative etiologies may allow for timely escalation of therapy and prevention of severe complications.

In patients with persistent gastrointestinal symptoms, including chronic diarrhea, unexplained weight loss, and abdominal pain, early endoscopic evaluation with intubation of the terminal ileum and biopsy is essential for accurate diagnosis [9]. Endoscopic appearance alone may be insufficient to distinguish intestinal Behçet’s disease from IBD, particularly when mucosal findings overlap. Histopathologic assessment, therefore, plays a central role in clarifying the underlying disease process and guiding management.

In the context of this case, histologic findings were essential in guiding the immunosuppressive strategy. The empiric treatment for presumed Behçet’s disease provided partial symptom relief but failed to control the underlying transmural intestinal inflammation. In contrast, Crohn’s disease requires targeted therapy directed at the transmural disease activity. Reliance on clinical features alone, without histologic confirmation, may result in under-treatment and delayed initiation of disease-modifying therapies.

Management of Crohn’s disease is guided by disease severity and risk stratification. For patients with moderate-to-severe or high-risk disease, early initiation of biologic therapy is the standard of care. Anti-TNF agents, such as infliximab, are the most effective therapies for inducing and maintaining remission and achieving mucosal healing. Corticosteroids are reserved for short-term induction only, while aminosalicylates have no established role in the treatment of active Crohn’s disease [10]. At the most recent follow-up, the patient remains under active gastroenterology care and continues maintenance therapy with infliximab. He has demonstrated sustained clinical improvement, with resolution of abdominal pain and diarrhea, progressive weight gain, and normalization of inflammatory markers. He has experienced no recurrent hospitalizations or disease flares since initiation of biologic therapy. The patient continues to live with a functioning ostomy and has not yet undergone reversal. Overall, his clinical course reflects a favorable response to targeted biologic therapy with ongoing functional and nutritional recovery.

Finally, this case underscores the importance of multidisciplinary collaboration in the management of complex inflammatory conditions. Coordinated involvement of rheumatology, gastroenterology, pathology, and surgery was critical to establishing the correct diagnosis and guiding appropriate therapy. Integration of medical, surgical, and diagnostic expertise facilitates timely reassessment and optimizes patient outcomes [11].

## Conclusions

Crohn’s disease and Behçet’s disease may present with overlapping mucocutaneous and gastrointestinal manifestations, creating substantial diagnostic complexity in clinical practice. This case underscores the importance of maintaining diagnostic vigilance, timely endoscopic evaluation with histologic confirmation, and multidisciplinary collaboration in patients with persistent or progressive inflammatory symptoms. Coordinated involvement of rheumatology, gastroenterology, pathology, and surgery was critical to establishing the correct diagnosis and guiding appropriate therapy. Early recognition of high-risk disease features and prompt initiation of biologic treatment are essential to preventing irreversible complications and optimizing long-term outcomes.
